# Contextual cues can be used to predict the likelihood of and reduce interference from salient distractors

**DOI:** 10.3758/s13414-024-03004-3

**Published:** 2025-01-10

**Authors:** Jeff Moher, Andrew B. Leber

**Affiliations:** 1 Psychology Department, Connecticut College, 270 Mohegan Avenue, New London, CT 06320 USA; 2 Department of Psychology, The Ohio State University, 1835 Neil Ave, Columbus, OH 43210 USA

**Keywords:** Tentional capture, Precuing, Attention and memory

## Abstract

Our attention can sometimes be disrupted by salient but irrelevant objects in the environment. This distractor interference can be reduced when distractors appear frequently, allowing us to anticipate their presence. However, it remains unknown whether distractor frequency can be learned implicitly across distinct contexts. In other words, can we implicitly learn that in certain situations a distractor is more likely to appear, and use that knowledge to minimize the impact that the distractor has on our behavior? In two experiments, we explored this question by asking participants to find a unique shape target in displays that could contain a color singleton distractor. Forest or city backgrounds were presented on each trial, and unbeknownst to the participants, each image category was associated with a different distractor probability. We found that distractor interference was reduced when the image predicted a high rather than low probability of distractor presence on the upcoming trial, even though the location and (in Experiment 2) the color of the distractor was completely unpredictable. These effects appear to be driven by implicit rather explicit learning. We conclude that implicit learning of context-specific distractor probabilities can drive flexible strategies for the reduction of distractor interference.

**Supplementary information:**

The online version contains supplementary material available at 10.3758/s13414-024-03004-3.

Our visual attention is not fully under our control. Sometimes an object can capture our attention due to its physical properties, even when that object is seemingly irrelevant to the task at hand. Perceptually salient distractor objects, for example, can capture attention and disrupt task performance under some circumstances. One classic approach to studying distractor interference is the additional singleton paradigm (e.g., Theeuwes, 1992). In this task, observers search for a unique target shape while on some trials one of the nontargets is presented in a different color. This singleton color distractor can produce attentional capture, evidenced by increased response times to the target when a distractor is present, and eye movements directed to the distractor’s location (e.g., Theeuwes, 1992; Theeuwes et al., 1998), though there is considerable debate about the extent to which the impact of salient distractors on task performance is automatic or can be minimized or eliminated (see, e.g., Luck et al., 2021, for a review).

One factor that can change how a salient distractor disrupts attention is the frequency with which distractors appear in a given task. For example, Geyer et al. (2008) varied the probability of distractor presence across blocks in the additional singleton paradigm. They found that distractor interference was reduced in blocks where the distractor probability was high (80%) compared with when it was low (20%). The authors concluded that when the incentive to suppress distractors is high because distractors appear often, the impact of those distractors is reduced through top-down attentional control (see also, e.g., Crump et al., 2018; Müller et al., 2009), though other authors used similar results to argue that statistical learning (e.g., Sayim et al., 2010; Valsecchi & Turatto, 2021) or proactive distractor suppression (e.g., Won et al., 2019) are involved in the reduction of distractor interference when distractor probability is high.

A challenge involved in manipulating the frequency of distractor presence across blocks is that doing so changes the frequency of certain intertrial contingencies, such as the repetition of distractor presence across consecutive trials. For example, for any given distractor-present trial, if the block-wise probability of distractor presence is 80%, the probability that the preceding trial also included a distractor is higher than if the block-wise distractor probability is 20%. These factors have previously been shown to impact behavior, with the typical observation that distractor interference is reduced when a distractor is present on consecutive trials (e.g., Kumada & Humphreys, 2002), similar to how conflict has a reduced impact in cognitive control tasks, such as the Stroop task, if conflict was also present on the prior trial (e.g., Gratton et al., 1992).

A similar issue of intertrial contingencies arose in studies examining the probabilistic cueing of target locations. Walthew and Gilchrist (2006) found that probabilistic learning benefits for high-probability target locations were eliminated if the trials were constrained such that the target’s location never repeated on consecutive trials. Jones and Kaschak (2012), however, were able to find evidence of global probability learning even in the absence of trial-to-trial repetitions of target location. Jiang et al. (2013) further demonstrated that the prioritization of locations where targets occur frequently persists even across new contexts where that location is no longer a frequent target location. The authors of the studies referenced above involving distractor probability all examined intertrial effects, finding in each case that intertrial contingencies did not explain the entirety of the observed effects of distractor probability on behavior. However, these intertrial contingencies were still present in each of these distractor-probability experiments and did, in most cases, influence behavior, making it difficult to isolate or remove their effects entirely.

Thus, the question remained whether distractor probabilities can influence attention when these intertrial contingencies are eliminated. To address this, Moher et al. (2011) used a novel approach of cueing distractor probability on a trial-by-trial basis. This method has the advantage of equating across conditions the probability for any given trial that a distractor appeared on the preceding trial, as well as equating the conditional probability that a distractor location is repeated across conditions. Moher et al. found that eye movements to distractors were less frequent when the pretrial cue indicated that distractor probability was high rather than low, suggesting that distractor probabilities can affect behavior when intertrial contingencies are equated across conditions. These results also demonstrate that distractor probability information can be flexibly used in order to prepare for distractors on a moment-to-moment basis.

A key difference between Moher et al. (2011) and prior studies was that distractor information was explicitly cued. The strategy that was used by participants likely involved an explicit, deliberate attempt to avoid capture from the salient distractor. Thus, an open question remains: Can distractor probabilities be learned, and distinct distractor handling strategies employed in a flexible and moment-to-moment manner, based on implicit learning? In the present study, we explore this question using contextual task cues to indicate distractor probability.

Context has long been known to influence cognition (e.g., Gershman et al., 2010) and can specifically influence target selection in visual search (Chun & Jiang, 1998). Cosman and Vecera (2013) developed a novel paradigm to explore the role that contextual learning plays in visual search when salient distractors were present. In that study, participants learned to associate specific search strategies (singleton detection mode or feature search mode; cf. Bacon & Egeth, 1994) with specific background image categories (cities and forests). Then, observers were given subsequent “option” trials in which either search strategy was available, and either a city or forest background was present on each trial. The authors found that participants used search strategies that matched the image associations they had learned. For example, if a participant learned to use singleton detection mode with forest backgrounds, they were more likely to implement a singleton detection mode strategy on subsequent “option” trials with forest backgrounds.

Recently, Allon and Leber (2024) used a similar approach to examine whether contextual learning could be used to learn probabilities of spatial distractor locations. Previous research has established that participants can learn when distractors are more likely to appear at certain locations, and use that information to minimize the impact of those distractors (e.g., Goschy et al., 2014; Leber et al., 2016; Wang & Theeuwes, 2018). Similar to distractor probability manipulations, however, these designs often run into issues with unequal intertrial contingencies across different key experimental conditions.

Allon and Leber (2024) used an approach similar to Cosman and Vecera (2013) to avoid this issue, by associating different backgrounds with increased probabilities of distractors appearing at specific locations, and having background trial types, randomly intermixed. When they used background images alone to convey distractor location probability, background scenes had minimal impact on performance. Allon and Leber reasoned that participants were likely not attending the background, and thus learning was not taking place, similar to prior research suggesting that learning statistical regularities requires participants to direct attention toward the relevant to-be-learned components (e.g., Jiang & Chun, 2001; Turk-Browne et al., 2005). Thus, to ensure that participants attended the background, Allon and Leber added several additional elements to the design. The background image, in addition to being associated with different distractor location probabilities, also cued the reported feature of the target. In other words, a participant might indicate the orientation of a line inside the target if the background is a forest, while they might indicate which side of the target is chipped if the background is a city. To further distinguish the two task contexts, the authors also paired distinct sets of shapes with the forest and city backgrounds. Participants were able to learn to associate backgrounds with different distractor location probabilities, and use this information to minimize the impact of distractors at high probability locations. Furthermore, participants were largely unaware of the relationship between background and distractor location probability at the end of the experiments, suggesting that contextually cued strategy shifts were an implicit rather than explicit process on the part of the observers. Other recent research by Turatto and colleagues (e.g., De Tommaso et al., 2024; Turatto et al., 2018) has similarly shown evidence for contextual learning of distractor suppression for distractors at specific locations.

In the present study, we adapted the Allon and Leber (2024) approach to manipulate distractor probability across different task contexts. One background image category was associated with high distractor probability, while the other background image category was associated with low distractor probability. If participants are able to learn these associations and implement flexible strategies based on this knowledge, we expect that distractor interference will be reduced when the background predicts high rather than low distractor probability. Furthermore, if this learning and strategy adjustment is implicit, we expect participants to demonstrate very little explicit knowledge at the end of the experiment when asked about the relationship between backgrounds and distractor probability.

## Experiment 1

### Methods

We collected data online from 89 participants at Prolific.co and through the Connecticut College subject pool (mean age = 24.4 years, 45 women, 41 men, one genderqueer, and two did not report gender). Because of the difficult nature of the task, we anticipated that some participants would struggle with the instructions, and thus their data would not be able to provide insight into the learning of the relationship between context and distractor probability. Therefore, we applied an exclusion criterion that participants must be at least 80% accurate over the course of the task. With this criterion applied, 38 participants were removed from analysis. The mean accuracy of these removed participants was 62%.^1^ Participants were required to have normal or corrected-to-normal color vision. All protocols were approved by the institutional review boards at Connecticut College and Ohio State University. Participants were given monetary compensation or course credit in exchange for completing the study. Methods for both experiments were preregistered online (https://osf.io/fqnue/), and all data are available at that site.

#### Stimuli

Custom software was created using JavaScript and adapted sample scripts from PsiTurk (Gureckis et al., 2016). Six background images were used in two categories: three forest images and three city images (the same images that were used in Allon & Leber, 2024). Each of these images, when present, were presented centrally at a size of 1,024 × 683 pixels. At the beginning of each trial, a black box measuring 350 × 350 pixels was presented at the center of the background image, containing a white fixation cross measuring 20 × 20 pixels that was presented at the center of the box. Subsequently, six shape outlines were presented in a ring surrounding fixation, with a center-to-center distance between fixation and each shape of approximately 155 pixels. One shape appeared at the 12:00 position, and the remaining shapes were equally spaced around the ring at 2:00, 4:00, 6:00, 8:00, and 10:00. Each shape was approximately 56 × 56 pixels. There were two possible combinations of shapes: circles and diamonds, or squares and pentagons. For each participant, one set of shapes was assigned to one background category, while the other set was assigned to the other background category. On each trial, one of the shapes was randomly chosen as the target shape, and one item would appear in this shape at a randomly selected location. The remaining five items would appear in the opposite shape of that pair, thus making the target a shape singleton. On distractor-present trials, one of the nontarget shapes at a randomly selected location would appear in a different color. For each participant, either blue or green was set as the default color for all objects. The other color was then used as the color for salient distractors on distractor-present trials.

Each shape had a gap measuring approximately 25 pixels, randomly selected to be on either the left or right side. Each shape also contained a line measuring 4 × 30 pixels, randomly chosen to be oriented either horizontally or vertically.

#### Procedure

**Fig. 1:**
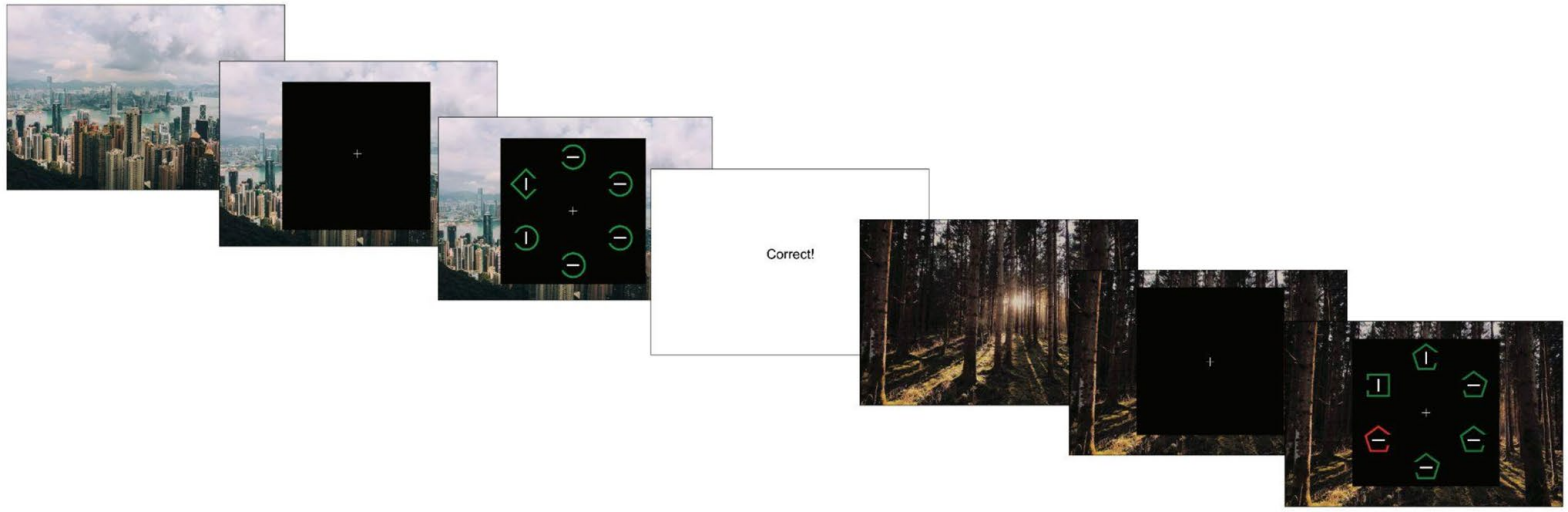
An example trial sequence from Experiment 1. Participants reported either the orientation of the line inside the unique shape or on which side a gap appeared on the shape, depending on which background image was present. Images not to scale. Underneath the display, a brief instruction appeared reminding the participants which reported feature was associated with the current background image type (e.g., “Z for gap on left, M for gap on right”). This is not shown in the image as the text would be too small to read

Participants were instructed that on every trial, there would be a unique shape target present. The response requirements differed depending on the background image category. For one background image category (forests or cities), participants were instructed to report which side of the shape had a gap with a key press. For the remaining background image category, participants were instructed to indicate whether the line inside the shape was horizontal or vertical with a key press. Which background went with which reported feature was randomly assigned for each participant. Background image categories were also associated with distractor probability. For one background image category, distractors were present on a randomly selected 20% of all trials. For the other background category, distractors were present on a randomly selected 80% of all trials. Which image category was associated with which distractor probability was randomly assigned for each participant. The order of all background images was randomly intermixed such that the background was unpredictable from one trial to the next. Participants were not explicitly informed of the relationship between background type and the probability of distractor presence (Fig. 1).

On each trial, the background image was presented for 1 s. After this, a fixation cross was presented for 1 s, followed by the trial display, which remained on-screen until participants made a response or until 5 s elapsed. At the bottom of the screen, on each trial, there was a written reminder of which target feature participants were supposed to respond to for that trial (gap or line orientation), and a reminder of which key went with which response. Following each trial, feedback was presented in the form of text on the screen that read either “correct!” or “incorrect” and remained on the screen for 1 s.

Following instructions, participants completed 20 practice trials. Following practice, participants completed four blocks of 100 trials each. At the end of the experiment, participants were presented with a series of questions to assess their explicit awareness of the relationship between background images and distractor probability. First, we indicated that we manipulated the probability of distractor presence such that distractors were more likely to appear in some backgrounds than in other backgrounds, and we asked participants if they noticed this relationship. Next, for each of the six images used, participants were asked to rate the likelihood that a distractor would appear on a trial in which that image was used as a background by moving a slider between 0 and 100, as well as to indicate how confident they were in their answer on a scale from 1 to 7. Finally, they were asked if they used any particular strategy or had any other comments about the experiment as an open-ended question.

### Results

We conducted 2 × 2 analyses of variance (ANOVAs), with the factors distractor probability (high or low) and distractor presence (present or absent) on error rate and response time (RT) dependent measures.

#### Error rate

**Fig. 2:**
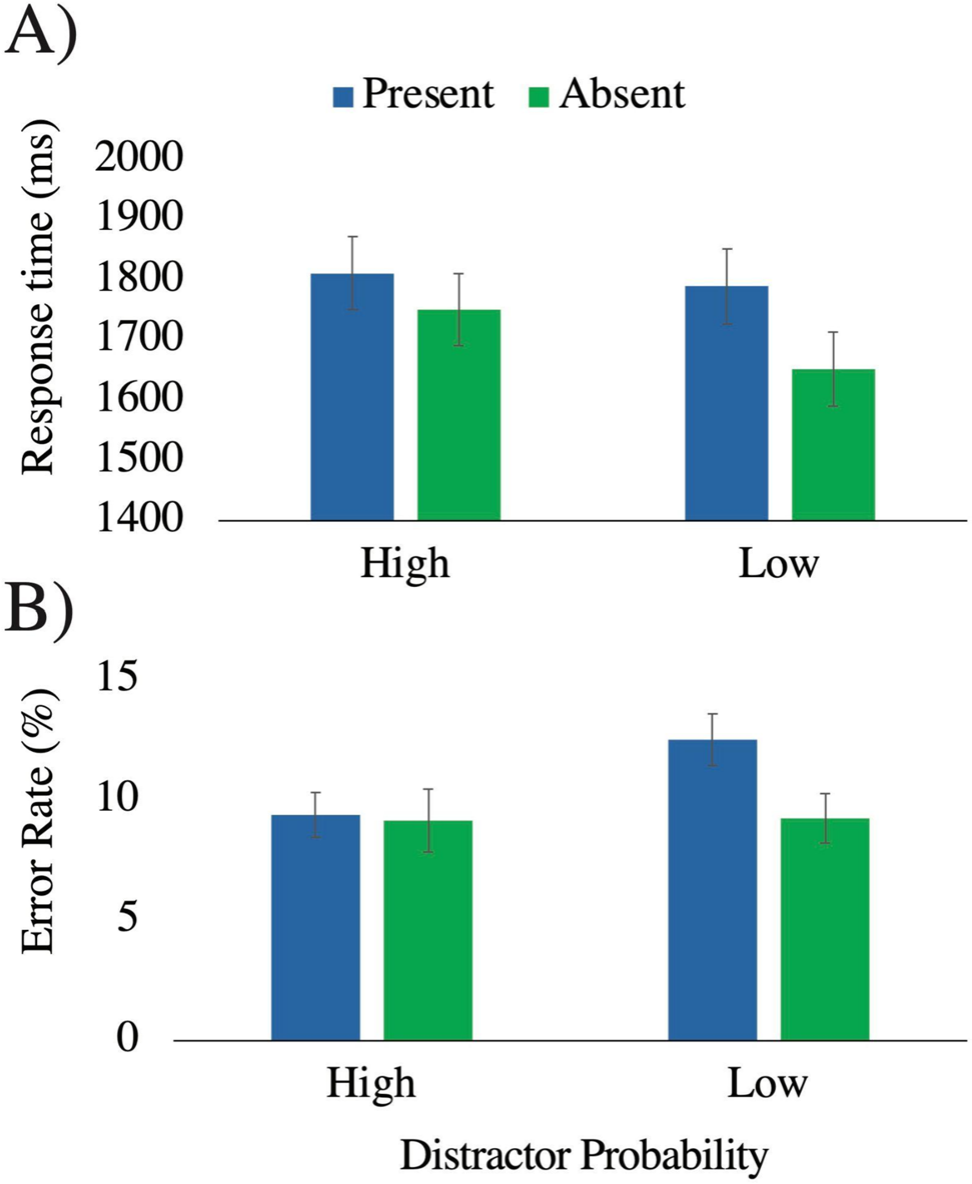
RT data **A)** and error rate data **B)** from Experiment 1, as a function of whether a distractor was present (blue) or absent (green), and whether distractor probability was high or low (*x*-axis). Error bars reflect standard error of the mean. (Color figure online)

There was a main effect of distractor presence on error rate, *F*(1,50) = 10.07, *p* = .003, η_p_^2^ = .17, mediated by a significant interaction between distractor probability and distractor presence, *F*(1,50) = 12.65, *p* < .001, η_p_^2^ = .20. Simple main effects analyses revealed that with high distractor probability backgrounds, there was no difference in error rate between distractor-present (9.4%) and distractor-absent (9.2%) trials, *F*(1,50) < 1, η_p_^2^ = .002. However, with low distractor probability backgrounds, error rate was higher when distractors were present (12.5%) compared with when they were absent (9.3%), *F*(1,50) = 22.60, *p* < .001, η_p_^2^ = .31 (Fig. 2). In other words, the distractor negatively impacted accuracy on the task, but only in the condition in which the background image indicated a low probability of distractor presence. There was no main effect of distractor probability, *F*(1,50) = 1.39, *p* = .24, η_p_^2^ = .03.

We conducted a second analysis with the additional factor of block (1, 2, 3, or 4) in order to determine whether the interaction between distractor presence and distractor probability changed over the course of the task, as participants might learn more about the relationship between context and distractor probability. There was a main effect of block, *F*(3,150) = 4.79, *p* = .003, η_p_^2^ = .09, with more errors on Block 1 (13%) compared with later blocks (Block 2: 8.6%, Block 3: 8.9%, Block 4: 10%). However, there were no two or three-way interactions with block, all *p *values > .65. The results of the full ANOVA are available in Table S1 in the Supplementary Materials.

#### Response time

We used a recursive trimming procedure to eliminate outlier RTs (Van Selst & Jolicoeur, 1994), resulting in the elimination of 0.7% of all trials. As with error rate, there was a main effect of distractor presence on RT, *F*(1,50) = 45.89, *p* < .001, η_p_^2^ = .48, moderated by a significant interaction, *F*(1,50) = 9.26, *p* = .004, η_p_^2^ = .16. There was a significant cost of distractor presence in the high probability condition (present: 1,812 ms, absent: 1,751 ms), *F*(1,50) = 10.36, *p* = .002, η_p_^2^ = .17. There was a much larger effect of distractor presence in the low probability condition (present: 1,789 ms, absent: 1,652 ms), *F*(1,50) = 48.57, *p* < .001, η_p_^2^ = .49. We also conducted simple main effects analysis to compare the effect of probability across distractor-present and distractor-absent trials separately; although the effect appeared to be driven largely by increased RTs on distractor-absent trials in the high probability condition, the effect of probability did not reach significance for either distractor-present or distractor-absent trials, *p* values > .15. There was no main effect of distractor probability on RT, *F*(1,50) < 1, η_p_^2^ = .02.

As with error rate, we conducted a second analysis with block as an additional factor. In this analysis, 0.8% of trials were eliminated as outliers through a recursive trimming procedure (Van Selst & Jolicoeur, 1994). There was a main effect of block, *F*(3,150) = 53.35, *p* < .001, η_p_^2^ = .52, with RTs decreasing over the course of the experiment. However, as with error rate, there were no two-way or three-way interactions between block and other factors, all *p* values > .62. The results of the full ANOVA are available in Table S1 in the Supplementary Materials.

#### Explicit knowledge

When participants were asked to estimate the probability that a distractor would appear with a given background image, they gave a higher estimate for high probability backgrounds (39.9%) compared with low probability background (33.8%), *t*(50) = 2.79, *p* = .007, Cohen’s *d* = 0.39. While this difference was statistically significant, it was nowhere near the true probabilities of the two conditions (80% and 20%).

Participants were also asked whether they explicitly noticed the relationship between background and distractor probability. Twenty-six participants said that they did notice a relationship, while 25 participants did not notice the relationship. We conducted a 2 × 2 ANOVA with a within-subjects factor of probability estimate (high or low) and a between-subjects factor of reported noticing of the relationship (yes or no). There was no interaction, *F*(1,49) < 1, η_p_^2^ = .001. This suggests that participants who reported noticing the relationship between distractor probability and background image were no better at estimating distractor probability compared with participants who did not report noticing this relationship.

As an exploratory analysis, we also conducted a correlation analysis comparing the magnitude of the interaction effect for each participant (for both error rate and RT, separately) with the magnitude of the difference in rated expected probability between high and low probability backgrounds. Neither correlation was significant, *p* values > .67, suggesting that the extent to which a participant estimated that distractors were more probable in the high probability condition at the end of the experiment did not have any relationship with how those backgrounds impacted attentional capture. We further conducted additional ANOVAs with distractor probability and distractor presence as within-subjects factors, and reported noticing as a between-subjects factor. For both error rates and RT, there were no three-way interaction, *F* values < 1, nor any other main effects or interactions with reported noticing as a factor, *p* values > .11, suggesting that whether participants reported noticing the relationship between distractor probability and background had no impact on their behavior.

To summarize Experiment 1, across measures of both error rate and RT, the impact of a salient distractor was more pronounced when background images indicated that distractor probability was low compared with when it was high. In other words, when participants were able to anticipate that a distractor was likely to appear, the impact of that distractor was diminished. Participants were able to distinguish between high and low probability backgrounds in a postexperiment survey to some extent, but their estimates of distractor probability were far removed from reality and the magnitude of their estimates did not correlate with performance on the task. Furthermore, participants who reported explicitly noticing the relationship between backgrounds and distractor probability were no better at making these estimates than participants who reported not noticing any relationship between background and distractor probability, and these two groups showed no differences in behavior during the actual task either. Together, these data suggest that participants are able to learn associations between contextual task cues and distractor probability, and use that information to flexibly and implicitly reduce the impact of distractors when they are likely to appear.

## Experiment 2

In Experiment 1, the color of the distractor was kept constant across display types for each participant. Thus, one possible explanation for the results of Experiment 1 is that participants are using a feature-based strategy when they anticipate a distractor is likely to appear, such as feature-based suppression (e.g., Moher et al., 2014; Stilwell et al., 2019; Vatterot & Vecera, 2012), or increased activation of the target feature (e.g., Egeth et al., 1984; Oxner et al., 2023). However, some recent studies have also found that distractor interference can be reduced even when the distractor color cannot be predicted. The existence of these “second-order suppression” (e.g., Gaspelin & Luck, 2018; Ma & Abrams, 2023; Won et al., 2019), also known as dimension-based suppression (e.g., Liesefeld & Müller, 2021; Müller et al., 2009; Sauter et al., 2018, 2019), suggests that people can minimize distractor interference using something other than a feature-based strategy, perhaps through inhibition of the strongest signal in a saliency map that is used to drive visual attention. The results of Experiment 1 do not distinguish between these two possible explanations of what participants were using to minimize distractor interference—a feature-based strategy or a second-order strategy.

In Experiment 2, to distinguish between these two accounts, the color of the shapes and a salient distractor if present were randomly selected on each trial. If participants can use a second-order strategy to minimize distractor interference, we expect to see similar results in Experiment 2 as we observed in Experiment 1. Alternatively, if participants were employing a feature-based strategy in Experiment 1, we expect the effects of background condition on distractor interference to shrink or disappear in Experiment 2, as the color of the distractor will be unpredictable from one trial to the next.

### Methods

We collected data online from 87 participants at Prolific.co and through the Connecticut College subject pool (mean age = 28.1 years, 39 women, 42 men, four genderfluid or nonbinary, and two did not report gender). With the same exclusion criterion applied as Experiment 1, 36 participants were removed from analysis. The mean accuracy of the removed participants was 58%.

All methods were identical to Experiment, 1 with the following exceptions. On each trial, the color of the displayed shapes was randomly selected to be either red, blue, yellow, or green. If a color singleton distractor was present, the color of the distractor was randomly selected to be one of the remaining three available colors. Colors were constrained such that the color of a distractor could not match the color of a distractor on the previous trial.

### Results

#### Error rate

**Fig. 3:**
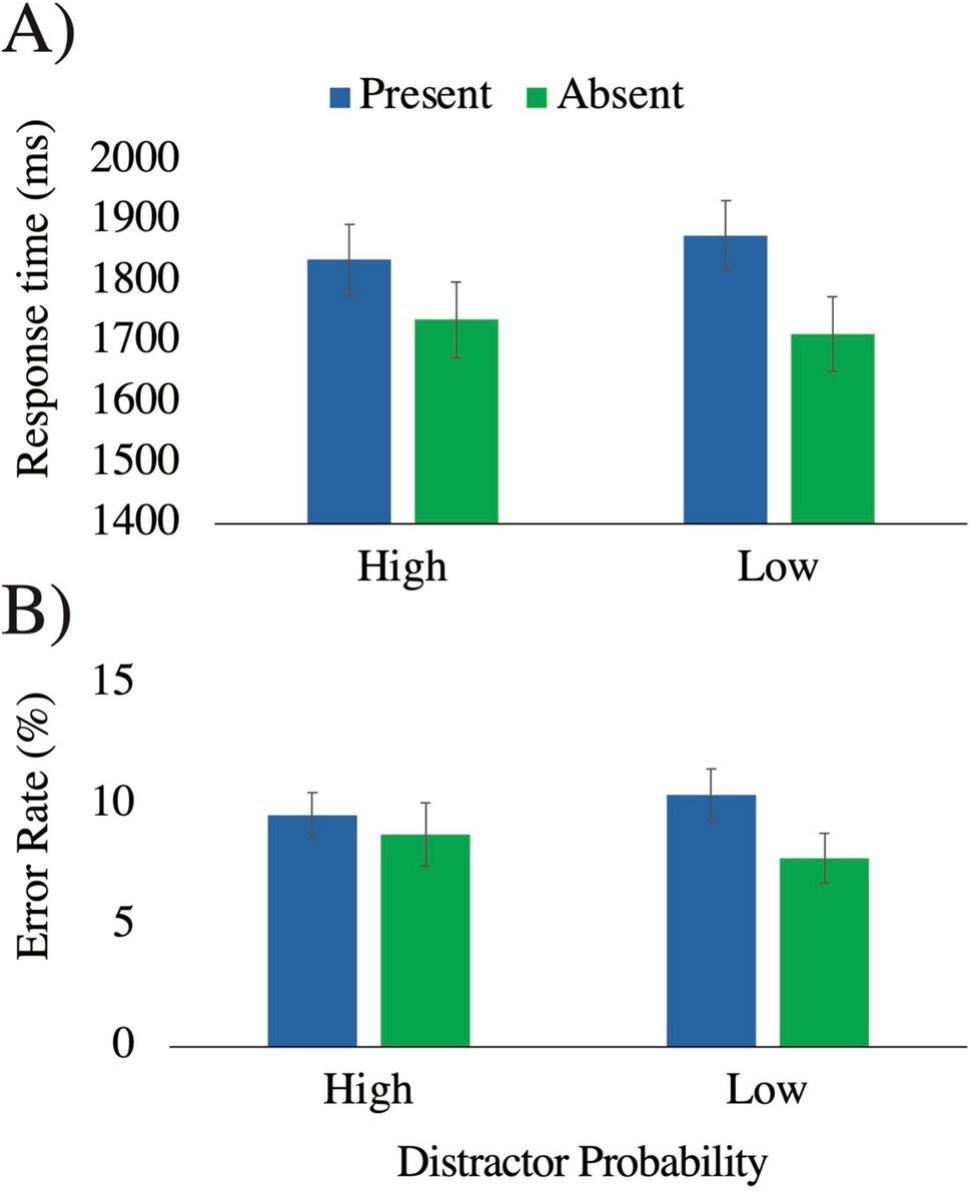
RT data **A)** and error rate data **B)** from Experiment 2, as a function of whether a distractor was present (blue) or absent (green), and whether distractor probability was high or low (*x*-axis). Error bars reflect standard error of the mean. (Color figure online)

A 2 × 2 ANOVA with the factors distractor probability and distractor presence revealed a main effect of distractor presence, *F*(1,50) = 11.19, *p* = .002, η_p_^2^ = .18, with higher error rates when distractors were present (10%) compared with when they were absent (8.3%). The interaction between distractor probability and distractor presence approached significance, *F*(1,50) = 3.68, *p* = .06, η_p_^2^ = .07. Simple main effects analyses revealed an effect of distractor presence in the low probability condition (present: 10.4%, absent: 7.8%), *F*(1,50) = 11.32, *p* = .001, η_p_^2^ = .19. There was no effect of distractor in the high-probability condition, *F*(1,50) = 1.83, *p* = .18, η_p_^2^ = .04 (Fig. 3). These effects mirror the results of Experiment 1. However, we note that these effects should be interpreted with caution given the marginal significance of the interaction effect. There was no main effect of distractor probability, *F*(1,50) < 1, η_p_^2^ < .001. As in Experiment 1, we also conducted a 2 × 2 × 4 ANOVA, with the additional factor of experimental block. There was a main effect of block, *F*(3,150) = 4.57, *p* = .004, η_p_^2^ = .08, with errors decreasing at later relative to earlier blocks. No other interactions with block were significant, all *p* values > .6. The results of the full ANOVA are available in Table S2 in the Supplemental Materials.

#### Response time

For RT, there was also a main effect of distractor presence, *F*(1,50) = 11.19, *p* = .002, η_p_^2^ = .18, moderated by a significant interaction, *F*(1,50) = 11.19, *p* = .002, η_p_^2^ = .18. As in Experiment 1, the magnitude of the distractor interference effect was larger in the low-probability condition (present: 1,877 ms, absent: 1,714 ms, η_p_^2^ = .57) compared with the high-probability condition (present: 1,837 ms, absent: 1,738 ms, η_p_^2^ = .36), and the effect of distractor presence was significant in both probability conditions, *p* values < .001 (Fig. 3). As in Experiment 1, we also conducted simple main effects analyses to examine the effect of probability across each level of distractor presence, and, as in Experiment 1, the effect of probability did not reach significance for either the distractor-present or distractor-absent condition, *p* values > .52. There was no main effect of distractor probability, *F*(1,50) < 1, η_p_^2^ < .001. In an additional ANOVA including block as a factor, there was a main effect of block, *F*(3,150) = 36.42, *p* < .001, η_p_^2^ = .42, with RTs decreasing in magnitude as time-on-task increased. However, block did not interact with any other factors, all *p* values > .09. The results of the full ANOVA are available in Table S2 in the Supplemental Materials.

Thus, once again, in Experiment 2, distractor interference was greater when the background image indicated that the probability of a distractor was low compared with when the background image indicated a high probability of distractor presence. This was true despite the fact that the distractor color was unpredictable from one trial to the next, providing evidence that context-driven reduction of distractor interference is implemented with a second-order strategy that does not depend on a feature-specific information.

#### Cross-experiment analyses

Patterns of behavior across both experiments appear similar. That is, in both experiments, the impact of a salient distractor is reduced, in measures of both error rate and RT, when the background indicates a high rather than low probability of distractor presence. To confirm this observation statistically, we conducted additional ANOVAs with within-subjects the factors distractor probability and distractor presence, the between-subjects factor experiment, and dependent variables error rate and RT. There was no main effect of experiment, nor did experiment interact with any other factors, in either ANOVA, all *p* values > .11. These cross-experiment analyses were not originally planned at the outset but were preregistered with Experiment 2. Post hoc power analyses revealed that the power to detect a significant effect of the magnitude observed here for the error rate interaction was only .16, and the power of the RT interaction was only .06. However, the power to detect a medium size effect (η_p_^2^ = .06) in the current data was .71 for error rate and for RT as calculated on MorePower 6.0.4 (Campbell & Thompson, 2012). Therefore, it seems unlikely that there is a medium or larger effect of keeping the distractor color consistent, but a small effect might be detected with a highly powered experimental design.

#### Explicit knowledge

As in Experiment 1, participants numerically rated high-probability background images as being more likely to predict a distractor-present trial (44.8%) than low-probability background images (41.8%); however, this did not reach statistical significance, *t*(50) = 1.33, *p* = .19, Cohen’s *d* = 0.19. Twenty-three participants reported noticing the relationship between background image and distractor probability, while 28 participants reported not noticing. As in Experiment 1, there was no interaction between probability rating and reported noticing, *F*(1,50) < 1, η_p_^2^ < .001.

Once again, we found no significant correlation between the magnitude of the rating difference and the magnitude of the interaction effect for either error rate or RT, *p* values > .13. We also conducted the same ANOVAs as Experiment 1 to explore whether reported noticing interacted with the other factors of interest in the primary analyses. Once again, we found no main effects or interactions with noticing as a factor, all *p* values > .07.

Across all of these analyses, there was no evidence in Experiment 2 that participants were explicitly aware of the relationship between background image and distractor probability, and no evidence that those who claimed to have noticed the relationship, or who reported a larger magnitude of difference between the two image types, showed different patterns of behavior compared with those who reported not noticing the relationship or showed smaller magnitude differences.

#### Exploratory target–distractor compatibility analysis

One possible explanation of the reduction in distractor interference in the high-probability background condition is that participants suppressed the salient distractor on trials in which they anticipated a distractor was likely to appear. It is difficult to directly test this possibility. However, in the current paradigm, we can explore one potential piece of evidence. On each distractor-present trial, the distractor had a reported feature that could either be compatible and match the reported feature of the target or be incompatible and cue the opposite response from the target (Theeuwes, 1996; Theeuwes & Burger, 1998). If participants are inhibiting the distractor in a reactive manner (see, e.g., Chang et al., 2023; Geng, 2014; Moher & Egeth, 2012), a predicted outcome might be that RTs are longer when the reported features of the target and distractors are compatible, because participants inhibit the reported feature of the distractor after an initial selection of that distractor. Note that this assumes participants can only identify the reported feature of an item when it is attended. While we think this is likely the case given the size of our stimuli, we cannot test this directly in the current experiment.

Combining data across both experiments, we conducted a 2 × 2 × 2 ANOVA with the within-subjects factors target–distractor compatibility on reported features (compatible vs. incompatible), and distractor probability, along with the between-subjects factor experiment. Note that only distractor-present trials were included in this analysis. We found a significant interaction between target–distractor compatibility and distractor probability, *F*(1,100) = 4.63, *p* = .03, η_p_^2^ = .04. On high distractor probability trials, there was a significant effect of target–distractor compatibility, *F*(1,100) = 13.82, *p* < .001, η_p_^2^ = .12, in which RTs were longer when the target and distractor were compatible (1,860 ms) compared with incompatible (1,814 ms). There was no significant effect of compatibility in the low-probability condition, *F*(1,100) < 1, η_p_^2^ = .003. No other main effects or interactions were significant, all *p* values > .13. A similar analysis was run on error rate data and found a main effect of probability, *F*(1,100) = 4.12, *p* = .045, η_p_^2^ = .04, reflecting higher error rates when distractor probability was low (11.4%) compared with high (9.5%). No other main effects or interactions were significant, *p* values > .19.

The RT results support the notion that the reduction in distractor interference observed on high-probability trials in both experiments is driven in part by reactive suppression of the salient distractor in the high-probability condition. This would also be inconsistent with a proactive suppression account (e.g., Gaspelin et al., 2015; Huang et al., 2021) for the current data, as it is unlikely that the reported feature of the distractor would have any effect if the distractor was not first selected given its small size. However, these results should be interpreted with caution given that they were the result of an exploratory analysis that was not part of our preregistration. Future experiments may further explore the underlying mechanisms involved in the reduction of distractor interference in the high-probability condition.

#### Exploratory intertrial analyses

As mentioned in the introduction, the experiment was designed to avoid extensive, cumulative intertrial effects, by interleaving the two different distractor probability conditions randomly. However, it is still possible that intertrial effects could be influencing behavior in the present task. For example, in prior studies, distractor presence on trial *N* − 1 reduced interference from a distractor on trial N (e.g., Müller et al., 2009; see also Kumada & Humphreys, 2002, for a location-specific version of this effect).

To see how intertrial contingencies affected attentional capture in the present studies, we conducted a 2 × 2 exploratory ANOVA with the factors distractor presence on the current trial and distractor presence on the previous trial, for both RT and error rate, with the data from both experiments included. There was no main effect of distractor presence on the previous trial for either measure, *p* values > .05. There was, however, a significant interaction for RT, *F*(1,101) = 11.17, *p* = .001, η_p_^2^ = .10. When a distractor was present on the previous trial, the distractor cost on the current trial was smaller (102 ms) compared with when no distractor was present on the previous trial (154 ms). For error rate, there was also a significant interaction, *F*(1,101) = 7.09, *p* = .009, η_p_^2^ = .07. Simple main effects revealed a similar pattern—when a distractor was present on the previous trial, there was no significant effect of distractor presence on the current trial (present: 9.3%, absent: 8.9%), *F*(1,101) = 0.35, *p* = .55. However, when no distractor was present on the previous trial, there were more errors when a distractor was present on the current trial (10.4%) compared with when no distractor was present on the current trial (8.3%), *F*(1,101) = 9.46, *p* = .003. This result is broadly consistent with prior studies in which the presence of a distractor on the immediately preceding trial reduces distractor interference on the current trial.

Given that we observed a significant effect of *N* − 1 distractor presence, one might question whether our main finding of an interaction between distractor probability and distractor presence is apparent on the first trial of a new context, or whether this effect is apparent only when there are a series of consecutive trials of the same context. In other words, can participants adapt their top-down set immediately upon seeing a new context, or are short-term learning or intertrial effects such as the one described above required to create this context-dependent change in distractor handling?

To examine this, we conducted a 2 × 2 × 2 exploratory analysis with the factors distractor presence, distractor probability, and context repetition (repeated vs. switched from the previous trial). Here, we focus only on reporting aspects of the analysis that deal with context repetition. RTs were shorter when the context was repeated (1,716 ms) compared with when it was switched (1,824 ms), *F*(1,101) = 119.00, *p* < .001, η_p_^2^ = .54. Errors were also reduced when context was repeated (8.1%) compared with when it was switched, (11.1%), *F*(1,101) = 66.32, *p* < .001, η_p_^2^ = .40.^2^ There was no significant interaction between context and any other factor for RT or accuracy, *p* values > .05. However, the three-way interaction for RT was marginally significant, *F*(1,101) = 3.90, *p* = .051, η_p_^2^ = .04.

To better parse this marginal interaction, for exploratory purposes, we conducted separate 2 × 2 ANOVAs, with the factors distractor presence and distractor probability for context-repeat and context-switch trials, focusing only on the interaction. For context-repeat trials, the interaction between distractor presence and distractor probability failed to reach significance, *F*(1,101) = 2.03, *p* = .16, η_p_^2^ = .02, though the pattern was the same that has been observed elsewhere in the paper, with a larger effect of capture on low probability trials (128 ms) compared with high probability trials (93 ms). For context switch trials, the interaction was significant, *F*(1,101) = 17.74, *p* < .001, η_p_^2^ = .15, with a much larger effect of capture on low-probability trials (176 ms) compared with high-probability trials (76 ms).

The main finding from these exploratory analyses is that context-driven effects are robust on the first trial following a context switch, as evidenced by the significant interaction between distractor presence and distractor probability on context-switch trials. If anything, context-driven effects on attentional capture are stronger in the first trial of a new contextual sequence. These data strongly suggest that the primary results reported in the two experiments are not simply due to a subset of trials where there are repetitions of context. Beyond this main finding we are hesitant to draw any additional inferences about the marginal three-way interaction or lack of a significant interaction between distractor probability and distractor presence on context repetition trials, based on the exploratory nature and the relatively few trials per condition in these analyses, as well as the complexity of the design.^3^

## General discussion

Across two experiments, we set out to determine whether implicit, contextual learning could support flexible changes in attentional strategy based on the expected probability that a salient distractor would appear. We found that when background images indicated a high probability that a salient distractor would appear on an upcoming trial, interference from that distractor was reduced. This was true in measures of both RT and error rate. Furthermore, this behavior did not appear to be driven by an explicit awareness, as evidenced by the lack of a relationship between answers on surveys related to explicit awareness and behavior on the main task. These results suggest that indeed participants can flexibly use implicit contextual cues to reduce distractor interference on a trial-by-trial basis when they expect a salient distractor to appear.

This outcome builds on prior work showing that knowledge of distractor probability can be used to reduce interference from frequently occurring distractors. Here, we have demonstrated that knowledge of distractor probability can be learned and implemented in a rapid, flexible, and implicit manner. Notably, this is likely a scenario that occurs in the real world with some frequency. That is, in our daily lives, we may implicitly learn that distractors occur more frequently in certain contexts (e.g., a particularly salient billboard on our morning commute), and learn to ignore those distractors in a flexible manner without the involvement of explicit awareness.

Similar to Allon and Leber (2024), we used multiple factors, including background image, shapes, and response requirements, to distinguish one task context from another. The downside to this approach is that we do not know the relative contribution of each context-related component to the observed effects. For example, the effects in the present study could be driven largely by associations between background images and distractor probability, or between shapes, or response requirements, or a combination of factors. Additional research is needed to distinguish among these possibilities. Still, regardless of which specific context-related feature is driving behavior, the present results do support that context-related learning of distractor probability influences distractor handling. Furthermore, Allon and Leber reasoned that this approach is not unlike real-world contexts in which changes in task context often involve multiple factors of the current task changing, rather than just the visual background. A similar logic applies to the present study.

Crump et al. (2018) used a modified version of the additional singleton paradigm to study similar questions related to context, attentional capture, and distractor probability (see also Thomson et al., 2014). Crump et al. employed two conditions—a congruent condition, in which the target was a color singleton, and an incongruent condition, in which a nontarget was a color singleton. They varied the proportion of congruent trials across different contexts, with context defined as either the particular set of shapes used for targets and distractors, or the location of all stimuli on the screen. The authors consistently found that distractor interference was reduced when contextual cues suggested a high probability of distractor presence, converging with similar approaches using cognitive control tasks such as the Stroop task in prior literature (see, e.g., Bugg & Crump, 2012, for a review).

The current study converges with these findings while also building in several key ways. One key difference between the current study and Crump et al. (2018) is that in our study, the salient object was never relevant. In Crump et al. (2018), it is likely that participants undergo a shift in the incentive to attend the salient item depending on the probability that the salient item is the target. In our study, no such strategy is likely to occur. Instead, participants always have an incentive to ignore the salient stimulus. Still, there were significant context-driven distractor probability effects, suggesting that the distractor processing changes depending on their expectations of distractor presence. A second key difference was that in our second experiment, we found that even when the color of the distractor was unpredictable, distractor interference was reduced when the context cued high distractor probability. This rules out the possibility of feature-based strategies as the driving force behind the reduction in distractor interference. Finally, across both experiments we observed impacts of contextually cued distractor probability on not only RT, but also response accuracy, suggesting that cues that indicate distractor probability can eliminate errors caused by task-irrelevant salient distractors.

On the other hand, our data contrast a recent study by Bogaerts et al. (2022). In that study, the authors explored whether second-order (or dimension-based) suppression of salient distractors was possible when distractors could be anticipated on a trial-by-trial basis. Using both implicit learning through predictable trial sequences, and explicit pretrial cues, the authors found no evidence of reduced capture when distractors could be anticipated. They concluded that trial-by-trial reduction in distractor interference based on expectations of distractor presence or absence is not possible. The present study provides evidence that it is indeed possible. What might have led to these discrepant results? One potential explanation is that the contextual cues used in the current study (the background images and the shapes paired with those images) provided more powerful cues than a repetition of trial sequences. Another possibility is that the longer RTs in the current study, which likely occurred due to complex task demands, provided more opportunity for a reduction in distractor interference. Still, further research will be needed to better understand when and how second-order strategies can be used to reduce distractor interference based on anticipated distractor presence.

Building on Allon and Leber (2024) and Cosman and Vecera (2013), the method used in the current study shows promise for understanding how observers might use flexible, dynamic strategies in dealing with salient distractors. By using randomly intermixed trial types, the method has a key advantage of minimizing differences in intertrial contingencies across conditions rather than trying to parcel them out with additional statistical analyses. Future research can use versions of this method to explore novel questions regarding what types of information can be learned implicitly, and subsequently used to inform cognitive strategies.

One important caveat to note in the present study is that in Experiment 1, the reduction of distractor interference in the high-probability condition was mostly driven by longer RTs in the distractor-absent condition, rather than a change in the distractor-present condition. Won et al. (2019) also observed a similar effect with a distractor probability manipulation, with longer RTs on distractor-absent trials in a high distractor probability condition. Experiment 2 appears to involve a mix of both of these changes, though notably, it is hard to parse this out statistically because in both experiments the effect of context failed to reach significance for either distractor-present or distractor-absent trials when examined separately. Why might this be the case? One explanation is that attempting to filter or ignore distractors is a resource-intensive process. Marini et al. (2013) argued this was indeed the case, and demonstrated that in instances where participants prepare for distraction, a large behavioral cost is seen if the expected distraction does not occur. Others have similarly argued that attentional resources are allocated ahead of expected distractions (e.g., Makovski, 2019). Something related may be at play in the current study. Preparation for distraction being a resource-intensive process would also help explain why participants are judicious in the use of their distractor-handling strategy, rather than implementing it on every trial regardless of whether a distractor is likely or not. That is, participants would likely minimize the use of cognitively effortful strategies unless they thought the use of such a strategy was likely to yield benefits. Prior research has found that participants do not always use optimal attentional control strategies (e.g., Irons & Leber, 2016), and it has been argued that individuals may often be capable of ignoring distractors but avoid the effort associated with implementing such strategies (e.g., Bacon & Egeth, 1994; Egeth et al., 2010; Leber, 2021).

A second possible explanation of the longer RTs on distractor-absent trials in the high-probability condition is that observers engaged in a strategy of delaying the start of the search process in order to avoid the short-lived but powerful initial wave of salience that drives early attentional selection (e.g., Donk & van Zoest, 2008). In any case, critically, the strategy used in the high-probability condition was without benefit, as accuracy costs produced by distractors were eliminated in the high-probability condition in both experiments.

We conducted an exploratory analysis to examine the mechanisms involved in the reduction of distractor interference in the high-probability condition. These analyses suggested a potentially reactive suppression mechanism that was implemented on high-probability distractor trials, based on an observed decrease in response compatibility costs in the high-probability condition. However, more data are needed to better understand what causes the difference in behavior on high and low distractor probability trials. In future studies, we plan to track eye movements in order to explore the time-course of attentional selection in this task and to better understand how the impact of distractors is minimized on high distractor probability trials.

We used a self-report measure at the end of the experiment to test explicit awareness of the link between distractor probability and background scenes. Although this analysis demonstrated little evidence of explicit awareness, it is possible that other measures might be more sensitive to probing participants’ knowledge of the link between task context and distractor probability. Recent evidence from other paradigms suggests that self-report measures can often underestimate actual awareness (e.g., Nartker et al., 2021). In our study, participants may have been responding cautiously by aiming toward the center of the scale, and they may have been confused by the presentation of each of the six backgrounds as separate questions, rather than being asked about image categories. In future studies, we plan to explore alternative approaches for measuring explicit awareness of the relationship between task context and distractor probability.

In sum, across two experiments we showed that distractor interference can be reduced when background images cue a high probability of salient distractor presence. These effects were observed across multiple dependent measures, and the strategies that observers used to reduce distractor interference on high probability trials appeared to be learned and implemented without explicit awareness. Together, these results suggest that humans are able to implicitly learn the relationship between contexts and distractions, and use this information flexibly to minimize distractor interference in contexts where frequent distractions occur.

## Supplementary information

Below is the link to the electronic supplementary material.

Supplementary file1 (PDF 233 KB)

## Data Availability

Methods for both experiments were preregistered online (https://osf.io/fqnue/), and raw and processed data are available at that site.

## Footnotes

1 An error was made in which one of the instruction pages gave the wrong key mapping for which target feature was associated with which response. This error was noticed early during data collection. Because there was practice with feedback, and correct instructions for key mapping were provided at the bottom of the display on every trial, and we used exclusion criteria to eliminate participants performing below 80%, these data were still included in all analyses.

2 It is important to note here that shape repetitions were possible only on context repetition trials because different sets of shapes were used in each context. As a result, when the context repeats, there is a 50% chance that the target shape also repeats, which we would expect to produce shorter RTs. When the context switches, there is a 100% chance that the target shape switches.

3 Numerous intertrial contingencies such as target shape repetition, response type repetition, and distractor presence on the previous trial, appeared at different frequencies in these different conditions. This creates discrepancies in the frequency of intertrial repetitions that are known to impact behavior, as well as creating the possibility of partial repetitions (e.g., Hommel, 1998; Lamy et al., 2010), further complicating interpretation of the data.

## Code availability

Data analysis code is available online (https://osf.io/fqnue/).
