# Supplementary material for: Contextual cues can be used to predict the likelihood of and reduce interference from salient distractors
Source: Atten Percept Psychophys. 2025 Jan 10;87(2):303–15. doi: 10.3758/s13414-024-03004-3 (PMC11865179; doi:10.3758/s13414-024-03004-3)
Supplement: Supplementary file 1 — Supplementary file1 (PDF 233 KB) [file 13414_2024_3004_MOESM1_ESM.pdf]

Supplementary Materials

Table S1

Outcome of three-way ANOVA from Experiment 1

Analysis	Dependent Variable	F	p	η_p^2
Distractor Presence (1,50)				
	RT	51.51	<.001	.51
	Error rate	4.90	.03	.09
Probability (1,50)				
	RT	0.86	.36	.02
	Error rate	0.81	.37	.02
Block (3,150)				
	RT	53.35	<.001	.52
	Error rate	4.79	.003	.09
Distractor x Probability (1,50)				
	RT	6.69	.01	.12
	Error rate	11.12	.002	.18
Distractor x Block (3,150)				
	RT	0.59	.62	.01
	Error rate	0.54	.66	.01
Probability x Block (3,150)				
	RT	0.18	.91	<.01
	Error rate	0.40	.76	.01
Three-way interaction (3,150)				
	RT	0.12	.95	<.01
	Error rate	0.12	.95	<.01

Table S2

Outcome of three-way ANOVA from Experiment 2

Analysis	Dependent Variable	*F*	*p*	η_p^2
Distractor Presence (1,50)				
	RT	100.68	<.001	.67
	Error rate	10.90	.002	.18
Probability (1,50)				
	RT	< .01	.97	<.01
	Error rate	< .01	> .99	< .01
Block (3,150)				
	RT	36.42	<.001	.42
	Error rate	4.57	.004	.08
Distractor x Probability (1,50)				
	RT	2.00	.16	.04
	Error rate	3.62	.06	.07
Distractor x Block (3,150)				
	RT	0.80	.49	.02
	Error rate	0.19	.90	<.01
Probability x Block (3,150)				
	RT	1.22	.30	.02
	Error rate	0.61	.61	.01
Three-way interaction (3,150)				
	RT	2.17	.09	.04
	Error rate	0.38	.77	.01
